# Effect of a Mating Type Gene Editing in *Lentinula edodes* Using RNP/Nanoparticle Complex

**DOI:** 10.3390/jof10120866

**Published:** 2024-12-13

**Authors:** Minseek Kim, Minji Oh, Ji-Hoon Im, Eun-Ji Lee, Hojin Ryu, Hyeon-Su Ro, Youn-Lee Oh

**Affiliations:** 1 Mushroom Science Division, National Institute of Horticultural and Herbal Science, Rural Development Administration, Eumseong 27709, Republic of Korea; kmstaur@gmail.com (M.K.); minji1228@korea.kr (M.O.); jihooni24@korea.kr (J.-H.I.); ejg1105@korea.kr (E.-J.L.); 2 Department of Biology, Chungbuk National University, Cheongju 28644, Republic of Korea; hjryu96@cbnu.ac.kr; 3 Department of Bio and Medical Bigdata (BK21), Research Institute of Life Sciences, Gyeongsang National University, Jinju 52828, Republic of Korea; rohyeon@gnu.ac.kr

**Keywords:** CRISPR/Cas9, DNA-free, RNP, nanoparticle, homeodomain, mating type

## Abstract

Gene editing using CRISPR/Cas9 is an innovative tool for developing new mushroom strains, offering a promising alternative to traditional breeding methods that are time-consuming and labor-intensive. However, plasmid-based gene editing presents several challenges, including the need for selecting appropriate promoters for Cas9 expression, optimizing codons for the Cas9 gene, the unintended insertion of fragmented plasmid DNA into genomic DNA (gDNA), and regulatory concerns related to genetically modified organisms (GMOs). To address these issues, we utilized a Ribonucleoprotein (RNP) complex consisting of Cas9 and gRNA for gene editing to modify the A mating-type gene of *Lentinula edodes*. To overcome the challenges posed by the large size of the Cas9 protein, which limits its penetration through the protoplast membrane, and the susceptibility of sgRNA to degradation, we developed a nanoparticle complex using calcium phosphate and polyacrylic acid. This approach significantly improved gene editing efficiency. Consequently, we successfully edited the mating-controlling genes *hd1* and *hd2* in *L. edodes* and examined the effects of their disruption on mating. Disruption of the *hd1* gene, which is known to influence mycelial growth, did not significantly affect growth or mating. In contrast, editing the *hd2* gene disrupted mating with compatible partners, highlighting its critical role in the mating process. The RNP-based transformation technology presented here offers significant advancement over traditional plasmid-based methods, enhancing the efficiency of targeted gene modification while avoiding the insertion of foreign genetic material, thereby mitigating GMO-related regulatory concerns.

## 1. Introduction

Mushrooms, primarily members of the Basidiomycota phylum, play a crucial role as decomposers in the carbon cycle within ecosystems through their active decomposition activities. They are also key agricultural commodities, cultivated for both culinary and medicinal purposes for centuries. Strain improvement has traditionally relied on crossbreeding haploid strains to enhance productivity and quality. While this method is effective, it is often labor-intensive, time-consuming, and challenging to develop strains with the desired traits. To address these limitations, various molecular breeding tools have been explored in recent decades. However, due to the unique structural and genetic repair mechanisms in mushrooms, these efforts have not yet been widely successful. Because mushrooms possess multinucleate cells with two distinct mating types within a single cell [1], achieving a genetically modified strain through double-strand breaks (DSBs) and subsequent repair mechanisms—such as homologous recombination (HR) and non-homologous end joining (NHEJ)—is particularly challenging [2,3]. Moreover, unlike bacteria and yeast, mushrooms lack autonomously replicating plasmids, which results in their low efficiency in polyethylene glycol (PEG)-mediated transformation. The insertion of genes using a binary vector through *Agrobacterium tumefaciens*-mediated transformation (ATMT) also presents challenges, as foreign genes are randomly integrated into the host genome [4,5,6,7]. Furthermore, these methods face additional regulatory hurdles, as they are difficult to implement in commercial strain development due to concerns related to genetically modified organisms (GMOs).

The CRISPR/Cas9 gene-editing technology, which emerged in 2013 with two landmark studies targeting human cells, has since been applied to various eukaryotes [8,9,10]. Research into gene editing for mushrooms gained significant traction starting in 2016, when Agaricus bisporus var. bisporus was edited to knock out the *ppo* gene associated with browning, thus bypassing the GMO regulations in the United States [11]. The first documented case of gene editing in mushrooms was by Qin et al. in 2017, who disrupted the *ura3* gene in *Ganoderma lucidum* by introducing Cas9 and in vitro-transcribed gRNA into its protoplasts [12]. That same year, Sugano et al. reported the successful knockout of *gfp* in *Coprinopsis cinerea* using a plasmid containing both Cas9 and gRNA [13]. Since 2020, CRISPR/Cas9 has been applied to numerous basidiomycetes, including *Ceriporiopsis subvermispora* [14], *C. cinerea* [13], *Pleurotus eryngii* [15], *Pleurotus ostreatus* [16,17,18], *Flammulina filiformis* [19], *L. edodes* [20], *G. lucidum* [12,21,22,23], and *Cordyceps militaris* [24,25,26]. Most studies utilize plasmids carrying Cas9-gRNA complexes, which are transiently expressed inside the cells and degrade without integrating into the genome. Another approach directly introduces the Cas9-gRNA ribonucleoprotein (RNP) complex into mushroom protoplasts [27]. This method avoids the introduction of foreign genes, such as antibiotic resistance markers, helping to circumvent GMO regulations. However, while the absence of selection markers simplifies the process, it also makes identifying successfully edited cells more challenging if the gene editing efficiency is low. Therefore, achieving high editing efficiency remains critical.

In mushroom strain development, the mating type system is a key consideration, as it governs sexual reproduction and genetic diversity in fungi. Particularly in basidiomycetes, the mating type system is highly complex and diverse, typically determined by two or more unlinked genetic loci, A and B [28]. The A locus contains *hd1* and *hd2*, which encode subunits of homeodomain (HD) transcription factors [29]. Although they differ in size and sequence, they share conserved domains, including dimerization domains, nuclear localization signals, and DNA-binding domains [30]. Notably, their N-termini are highly variable, facilitating heterodimerization through polar and hydrophobic interactions while preventing homodimerization [30]. This multiallelic nature of the A mating type is observed in species such as *Flammulina velutipes* [31], *Sporidiobolus salmonicolor* [32], *Pleurotus djamor* [33], *L. edodes* [34,35,36], and *Volvariella volvacea* [37], in which N-terminal variations drive diversity. The resulting HD dimers regulate essential events during mating, such as nuclear pairing, clamp cell formation, septation, and nuclear division. They also control the expression of the clamp connection formation protein 1 (CLP1) gene, which facilitates the movement of dividing nuclei through clamp connections [38,39]. Additionally, they regulate the zinc finger protein 2 (ZNF2) gene, which is critical for hyphal development and for differentiation into dikaryotic cells, and the pheromone receptor induction A (*priA*) gene, which mediates hyphal fusion through pheromone signaling [40,41,42].

In this study, we further investigated the effects of editing not only the *hd1* gene but also the *hd2* gene in *L. edodes*, building upon prior research that focused on *hd1*. *L. edodes*, a widely cultivated and economically important edible mushroom, is known for its nutritional and medicinal properties. However, genetic improvement has been hampered by its complex mating system and the inefficiency of traditional breeding techniques. To address these challenges, we utilized nanoparticles composed of calcium phosphate and polyacrylic acid to enhance the efficiency of an RNP-based gene editing tool. Our analysis of the gene-edited strains revealed that HD2 plays a more critical role than HD1 in the mating process of *L. edodes*, significantly impacting key functions during mating. Notably, this finding highlights the potential to improve our understanding of the complex mating system and to relax mating restrictions through gene editing, thereby enabling the development of superior strains. The use of the RNP/nanoparticle complex not only enhances editing efficiency but also enables precise genetic modifications without introducing foreign DNA, accelerating the development of advanced *L. edodes* strains. This approach mitigates regulatory concerns related to GMOs while offering additional benefits, such as improved growth rates, higher yields, and greater resilience, ultimately contributing to more efficient cultivation and better product quality.

## 2. Materials and Methods

### 2.1. Strains and Culture Conditions

Monokaryotic strains of *L. edodes* were obtained from Professor Hojin Ryu at the Chungbuk National University and Professor Hyeon-Su Ro at the Gyeongsang National University. The strain Sanjo705-13 (SJ705-13) was provided by the Chungbuk National University, while Sanjo710-A1 (SJ710-A1) and Sanjo701-A5 (SJ701-A5) were provided by the Gyeongsang National University. Each strain was cultured at 25 °C on potato dextrose agar (PDA) or in potato dextrose broth (PDB, Difco, Franklin Lakes, NJ, USA).

### 2.2. gRNA and Vector Design

**Figure 1:**
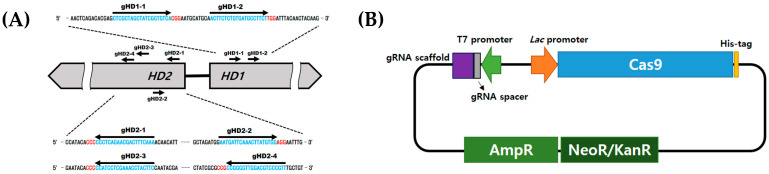
(**A**) Design of *hd1-* and *hd2*-targeted gRNA. The blue represents gRNA; the red represents the PAM sequence. (**B**) Schematic representation of the TA-V2-RNP vector for RNP production.

Two crRNAs targeting the *hd1* gene of *L. edodes* were provided by Professor Hojin Ryu at the Chungbuk National University [35]. Four crRNAs targeting the *hd2* gene were designed using CRISPOR version 5.2; http://crispor.tefor.net/ (accessed on 23 March 2022) (Figure 1A). Each gRNA was synthesized with tracrRNA and a T7 promoter, generating DNA fragments, which were then inserted into TA-V2 (Enzynomics, Daejeon, Republic of Korea) containing Cas9 at the 3′ end of the *Lac* promoter.

### 2.3. RNP Complex Purification

*E. coli* BL21 cells containing the TA-V2-RNP expression vector with each gRNA were cultured in LB broth (10 g/L tryptone, 5 g/L yeast extract, 10 g/L NaCl) supplemented with 50 μg/mL ampicillin at 25 °C, 180 rpm for 2 days. When OD_600_ reached 0.6, Cas9 expression was induced using 2 mM IPTG, and incubation continued for 16 h. The culture was centrifuged at 10,000× *g* for 15 min, and 30 mL of lysis buffer (20 mM Tris-HCl, 300 mM NaCl, 10 mM imidazole, pH = 8.0) was added to the pellet for resuspension. The suspension was sonicated using a VCX750 sonicator (Sonics, Newtown, CT, USA) and then centrifuged again. The supernatant was applied to a Ni-NTA agarose column (Qiagen, Hilden, Germany). The bound RNP was washed with washing buffer (20 mM Tris-HCl, 300 mM NaCl, 20 mM imidazole, pH = 8.0) and eluted with elution buffer (20 mM Tris-HCl, 300 mM NaCl, 250 mM imidazole, pH = 8.0). The eluate was dialyzed for 24 h in dialysis buffer (20 mM HEPES, 150 mM KCl, 10% glycerol, 1 mM DTT, 1 mM EDTA). The concentration of RNP was measured using the Bradford assay.

### 2.4. Evaluation of gRNA Efficiency Using In Vitro Cleavage Assay

The editing efficiency of each gRNA targeting *hd1* and *hd2* was assessed through an in vitro cleavage assay. Purified RNP was prepared at concentrations of 10, 100, and 1000 ng, and 1 μg of the PCR-amplified *hd1* and *hd2* fragments were incubated in reaction buffer (50 mM Tris–HCl, 0.1 M NaCl, 10 mM MgCl_2_, 1 mM DTT, pH = 7.9) at 37 °C for 20 min. The reaction mixture was analyzed through 1.2% agarose gel electrophoresis. The *hd1* and *hd2* DNA fragments were amplified using primer sets (Supplementary Table S1) under the following conditions: initial denaturation at 95 °C for 5 min; 30 s of denaturation at 95 °C, annealing at 57 °C for 30 s, and extension at 72 °C for 50 s for 25 cycles; and final extension at 72 °C for 5 min.

### 2.5. Formation of RNP/CaP Nanoparticle Complex and Protection Assay

This method was modified from Shuojun Li et al. [43]. RNPs (50 µg) were mixed with 183 µL of Buffer A (20 mM Tris-HCl, 300 mM NaCl, pH = 7.5) and vortexed. Then, 10.4 µL of 60 mM Na_2_HPO_4_ was added and vortexed again, followed by the addition of 25 µL of 100 mM CaCl_2_ and another vortex. After standing at room temperature for 5 min, the solution turned opaque. Subsequently, 5 µL of 1 M polyacrylic acid (PAA) was added, and the mixture was vortexed to improve the nanoparticle dispersion. The RNP/nanoparticle complex was washed three times with ddH_2_O, centrifuged at 10,000× *g* for 10 min, and resuspended in ddH_2_O, followed by ultrasonic dispersion. To test the protection capability, 25 µL of the formed RNP/Nanoparticle (NP) complex and 25 µL of RNP were treated with 3 µL of 10 mg/mL RNaseA (Bioneer, Daejeon, Republic of Korea) and incubated at 37 °C for 6 h. The nanoparticles were dissolved with 0.1 M HCl, and the solution was reacted with the *hd1* and *hd2* DNA fragments at 37 °C for 1 h. The samples were analyzed via electrophoresis on a 1.5% agarose gel.

### 2.6. Protoplast Preparation

The monokaryotic strain Sanjo701-13 was fragmented after 7 days of incubation at 25 °C on PDA medium. It was transferred to 150 mL of PDB for another 7 days under dark conditions. The cultured mycelium was filtered through Miracloth (Merck, Darmstadt, Germany) to remove the medium and washed with 0.6 M sucrose. The filtered mycelium was transferred to a 50 mL conical tube and treated with a mixture containing 10 mg/mL lysing enzyme (Sigma-Aldrich, St. Louis, MO, USA), 5 mg/mL Chimax-N (Amicogen, Jin-ju, Republic of Korea), 2 mg/mL cellulase (Sigma-Aldrich, USA), 1.5 mg/mL BSA (Sigma-Aldrich, USA), and 0.6 M sucrose. The mixture was incubated for 3 h at 25 °C in the dark. The reaction mixture was filtered through Miracloth, washed with 0.6 M sucrose, and centrifuged at 1600× *g* for 15 min at 4 °C, and the supernatant was removed. Protoplasts were suspended in 1 mL of STC buffer (10 mM Tris–HCl, 10 mM CaCl_2_, 0.6 M sucrose, pH = 7.4) and used for transformation. The number of protoplasts was counted using a hemocytometer (Marienfeld Superior, Lauda-Königshofen, Germany) and a microscope.

### 2.7. Transformation of L. edodes Using the RNP/Nanoparticle Complex

50 µL of each RNP/NP complex was mixed with 200 µL of the protoplast solution (final concentration: 1.0 × 10^7^ protoplasts) and 2 µL of Triton X-100. The mixture was incubated on ice for 40 min. After incubation, 1 mL of PTC buffer (containing 40% PEG4000 and STC buffer) was added, and the mixture was incubated at 25 °C for 20 min. The mixture was cultured on PDA medium containing 0.6 M sucrose at 25 °C for a week. The resulting colonies were transferred to fresh PDA plates for further cultivation.

### 2.8. DNA Extraction and RT-PCR Analysis

The transformants were cultured on PDA at 25 °C for 2 weeks, and the mycelium was collected and ground using liquid nitrogen. The genomic DNA was extracted using a genomic DNA prep kit (BIOFACT, Daejeon, Republic of Korea). To analyze the performance of the gRNA and verify the nanoparticle’s protective effect, PCR was performed using primer sets (Supplementary Table S1) for *hd1* and *hd2*. For mRNA expression analysis in the transformants, the total RNA was extracted from ground mycelium using the RNeasy Plant Mini Kit (Qiagen, Germany), and reverse transcription PCR (RT-PCR) was performed using pTOP RT-PCR kit (Enzynomics, Republic of Korea). The primer sets used for RT-PCR are listed in Supplementary Table S1. The relative expression of each target gene was assessed by comparing the band density of the triplicated samples against β-tubulin.

### 2.9. Mating Assay of Monokaryotic Strain

Each monokaryotic mycelium and transformant was inoculated 0.5 cm apart in the center of a PDA plate. After 2 weeks of incubation, the formation of clamp connections was observed using an optical microscope, and mating was confirmed using the A mating type marker (Supplementary Table S1).

## 3. Results

### 3.1. Transformation of L. edodes Using RNP/Nanoparticle Complex

**Figure 2:**
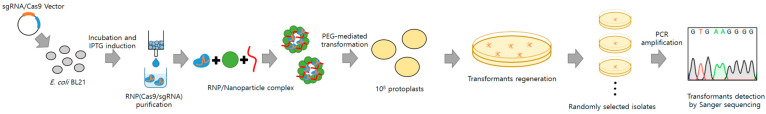
Summary diagram of the *L. edodes* transformation experiment using an RNP/NP complex. is study assembled a vector to simultaneously produce Cas9 and gRNA and used calcium phosphate (CaP) to form an RNP/nanoparticle complex (RNP/NP) to enhance efficiency and stability. The complex was introduced into *L. edodes* protoplasts through PEG-mediated transformation, and the transformants were analyzed via sequencing.

Unlike conventional gene-editing methods using RNP gene scissors, this study assembled the vector to simultaneously produce Cas9 and gRNA in the Cas9 production vector (Figure 1B). When IPTG was treated in *E. coli* BL21 strains, Cas9 and gRNA were overproduced together, resulting in a complete RNP complex. The produced RNP was extracted using a Ni-NTA column. To maximize efficiency, calcium phosphate (CaP), a material known for its pH responsiveness and high bioavailability, was used to form an RNP and CaP nanoparticle complex (RNP/NP). This combination enhanced the RNP cell membrane’s permeability and protected it from environmental factors. The formed RNP/NP complex was then introduced into the protoplasts of *L. edodes* through PEG-mediated transformation, and regenerated colonies were randomly selected and analyzed via sequencing to identify the transformants (Figure 2).

### 3.2. In Vitro Cleavage Assay for gRNA and Protection Assay for RNP/NP Complex

**Figure 3:**
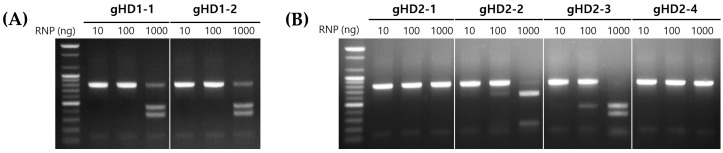
Cleavage of the *hd1* or *hd2* DNA amplicon by RNP complexes: (**A**) RNP complexes, gHD1-1 and gHD1-2, showed high cleavage activities to the HD1 DNA fragment. (**B**) RNP complexes harboring different gRNAs, gHD2-1, gHD2-2, gHD2-3, and gHD2-4, showed different cleavage activities to the HD2 DNA fragments. The RNP was incubated in different amounts (10, 100, and 1000 ng) with 1000 ng *hd1* or *hd2* DNA fragments.

The RNP complexes with gRNA1- and gRNA2-targeting *hd1*, gHD1-1 and gHD1-2, respectively, were reacted with the DNA fragment of *hd1*, confirming that both gRNAs exhibited nuclease activity (Figure 3A). When the RNP concentrations were set at 10, 100, and 1000 ng, clear activity was observed only at 1000 ng, with little to no activity at 10 and 100 ng. This indicates that sufficient activity can be obtained even when both Cas9 and sgRNA are simultaneously produced in *E. coli* and extracted in RNP form. The same method was used to confirm the nuclease activity of RNP complexes for *hd2*, including gHD2-1, gHD2-2, gHD2-3, and gHD2-4 (Figure 3B). Among the four RNP complexes, gHD2-1 and gHD2-4 showed no nuclease activity at any concentration, while gHD2-2 and gHD2-3 displayed high nuclease activity. Notably, unlike gHD1-1 and gHD1-2, nuclease activity was detected even at 100 ng.

**Figure 4:**
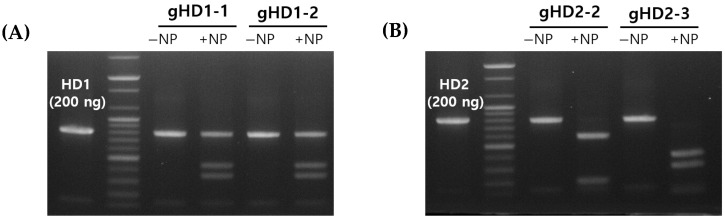
Protection of the RNP activity by nanoparticles against RNase A: (**A**) Cleavage of HD1 by RNP activity in the absence of or in the presence of nanoparticles (NP). (**B**) Cleavage of HD2 by RNP activity in the absence of or in the presence of nanoparticles with RNase A for 6 h at 37 °C.

To verify whether the CaP nanoparticles could protect sgRNA from environmental factors such as RNase, experiments were conducted by exposing both the RNP and RNP/NP complex to RNaseA. After a 6-h reaction, the structure of the RNP/NP complex was dismantled, and reactions with each target gene were performed (Figure 4A,B). The RNPs exposed to RNaseA lost all nuclease activity, while those complexed with CaP nanoparticles maintained their activity, confirming that the sgRNA was effectively protected.

### 3.3. Sequence Analysis of hd1 and hd2 Gene-Edited Transformants

**Figure 5:**
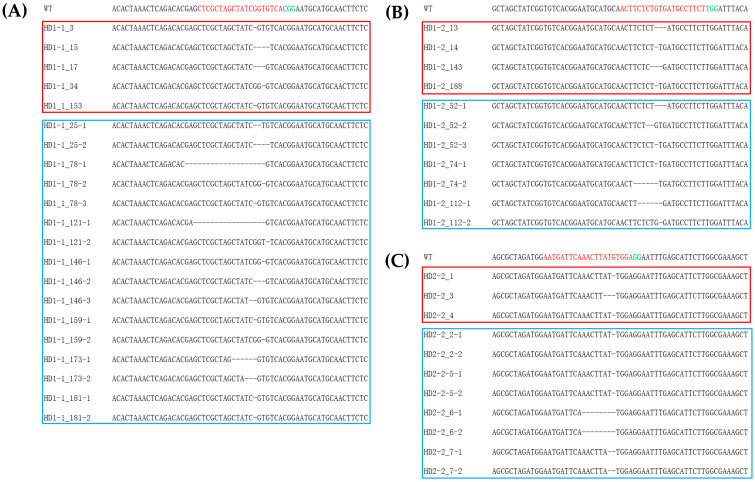
Sequence analysis of the *hd1* and *hd2* transformants: (**A**) Identification of five homokaryotic monokaryons and seven multinucleate monokaryons from the *hd1*-edited transformants with gHD1-1. (**B**) Identification of four homokaryotic monokaryons and three multinucleate monokaryons from the *HD1*-edited transformants with gHD1-2. (**C**) Identification of three homokaryotic monokaryons and four multinucleate monokaryons from the *hd2*-edited transformants with gHD2-2. The gRNA sequences are red-faced, while the PAM are in green. The red rectangle represents the sequence of homokaryotic monokaryons, and the blue rectangle represents the sequence of multinucleate monokaryons.

To evaluate the impact of the nanoparticles on transformation efficiency, the RNP/NP complex was independently introduced to the protoplasts through PEG-mediated transformation. A total of 200 regenerated colonies was randomly isolated and sequenced. The results showed that gHD1-1, gHD1-2, and gHD2-2 yielded 12, 7, and 7 transformants (Figure 5A–C), respectively. gHD2-3 failed to generate a transformant. Despite conducting experiments with a monokaryotic strain, 53.8% of the transformants contained complex sequences from two or more types. Specifically, the transformants HD1-1_25, HD1-1_121, HD1-2_74, and HD2-2_5 were found to contain not only the edited sequences but also the wild-type sequence. Analysis of the previously confirmed transformant sequences revealed a total of 21 single-base deletions, accounting for 50% of the total. Additionally, there were five cases of 2 bp deletions, seven cases of 3 bp deletions, two cases of 4 bp deletions, and seven cases with larger deletions. As a result, gene editing efficiencies of 6% for gHD1-1 and 3.5% for both gHD1-2 and gHD2-2 were observed.

### 3.4. Mating-Related Gene Expression in hd1 and hd2 Transformants

**Figure 6:**
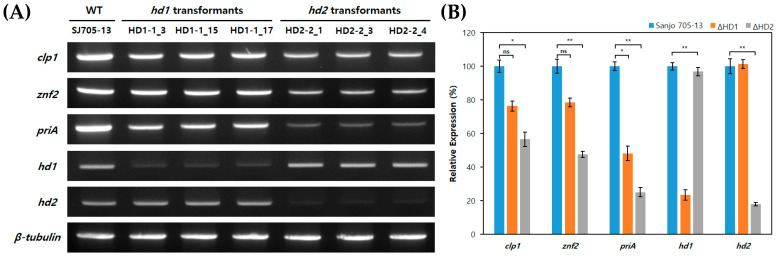
Analysis of the mRNA expression levels in the transformants: (**A**) RT-PCR was performed to analyze the mRNA expression levels of the *hd* genes and mating-related genes, with β-tubulin included as a loading control. (**B**) Comparison of the relative expression levels of the mating-related genes. The comparison was made based on the mRNA expression levels of the wild-type strain (Sanjo705-13). Error bars indicate the SE of the mean (*n* = 3; ns > 0.1 * *p* < 0.05, ** *p* < 0.01; Student’s *t*-test).

The expression levels of the genes regulated by the HD complex, such as *clp1*, *znf2*, and *priA* in the selected transformants and the wild-type strain SJ705-13, were evaluated by RT-PCR (Figure 6A). The expression levels of each gene showed consistent trends, regardless of the size of the deletion, and the impact was more significant in the *hd2* transformants as compared to the *hd1*. When comparing the relative expression levels of each gene against β-tubulin in the HD1-1_15 and HD2-2_3 transformants, HD1-1_15 showed no significant decrease in expression except for *priA* and the *hd1* gene (Figure 6B). In contrast, the *HD2* transformants showed reduced expression levels by more than half in all gene expressions except for the *hd1* gene.

### 3.5. Mating Analysis of hd1 and hd2 Transformants with Wild-Type Monokaryotic Strains

**Figure 7:**
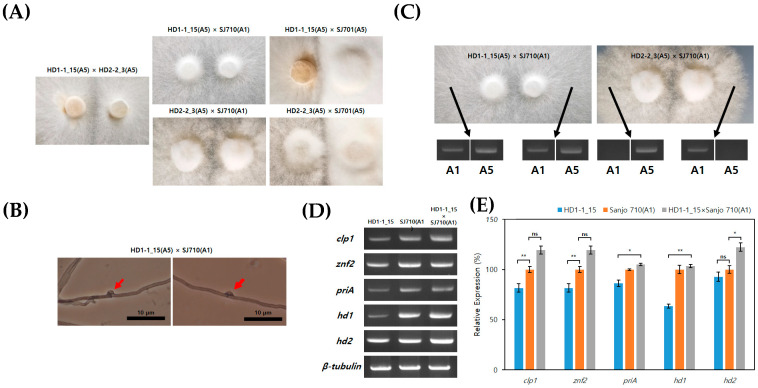
The impact of *hd1* and *hd2* gene deficiency on mating: (**A**) Mating assay between the wild-type monokaryotic strains SJ710 (A1) and SJ701(A5) and the transformants. Only the HD1-1_15×SJ710 (A1) combination resulted in the merging of mycelia. (**B**) Observation of clamp connection formation in the HD1-1_15 × SJ710 (A1) dikaryon. The red arrow indicates clamp connection formation. (**C**) Verification of mating in HD1-1_15 × SJ710 (A1) and HD2-2_3 × SJ710 (A1) using A1 and A5 A mating-type markers. (**D**,**E**) Analysis of mating-related gene expression levels using RT-PCR in monokaryotic strains HD1-1_15 and SJ710 (A1) and dikaryotic strain HD1-1_15 × SJ710 (A1). Error bars indicate the SE of the mean (*n* = 3; ns > 0.1 * *p* < 0.05, ** *p* < 0.01; Student’s *t*-test).

A mating assay was conducted to determine the impact of the *hd* gene-edited transformants on mating. The following pairings were tested: HD1-1_15 × HD2-2_3, HD1-1_15 × SJ710 (A1 mating type), HD1-1_15 × SJ701 (A5 mating type), HD2-2_3 × SJ710 (A1), and HD2-2_3 × SJ701 (A5) (Figure 7A). Only the pair of HD1-1_15 × SJ710 (A1) resulted in successful mating, while all other combinations failed to mate, showing barrage lines between the monokaryons. Observation under an optical microscope confirmed the formation of clamp connections only in the HD1-1_15 × SJ710 (A1) pair (Figure 7B). To verify nuclear migration, the nuclear types of mycelia ends in HD1-1_15 × SJ710 (A1) and HD2-2_3 × SJ710 (A1) were analyzed (Figure 7C). The fused mycelia of HD1-1_15 × SJ710 (A1) showed both A1 and A5 nuclei, confirming successful dikaryon formation. In contrast, HD2-2_3 × SJ710 (A1), which formed boundaries, showed only mononuclear types, indicating unsuccessful dikaryon formation. Finally, the gene expression levels of the successfully mated HD1-1_15 × SJ710 (A1) dikaryon were analyzed (Figure 7D,E). Despite the lower expression levels of the target genes in the *hd1* transformant as compared to the wild type, the dikaryon showed higher expression levels than the wild type, regardless of the transformant.

## 4. Discussion

Since 2020, research on gene editing in mushrooms has increased exponentially. Currently, most methods involve introducing plasmid DNA into protoplasts, but this approach remains challenging for species other than a few well-studied mushrooms. This is because, given the diversity of mushrooms, it is difficult to universally apply essential elements such as the selection of appropriate promoters for Cas9 and gRNA expression, codon optimization for Cas9, and the selection of suitable selectable markers. Additionally, when using plasmids, fragmented plasmid DNA has been reported to integrate randomly into the host’s genomic DNA [41].

To overcome these issues, DNA-independent gene editing has been attempted by directly introducing the Cas9-gRNA RNP complex into protoplasts of mushrooms such as *S. commune* [27], *C. cinerea* [44], *P. ostreatus* [45,46], and *G. lucidum* [47]. However, this method cannot use selectable markers like antibiotic resistance genes, as no foreign DNA is inserted. Therefore, the targets are limited to auxotrophic markers like *pyrG* and *leu2*, making selection difficult. Consequently, high-efficiency gene editing is essential, and to achieve this, this study utilized CaP nanoparticles [43].

Various RNP delivery platforms have been developed, such as cell-penetrating peptides, cationic lipids, gold nanoparticles, zeolitic imidazole frameworks (ZIFs), endosomal lysis agents, and receptor-mediated and endosomal escape agents like PEI [48,49]. However, these methods still face challenges related to RNP loading, release efficiency, simplicity, and stability, which limit their uses. In contrast, calcium phosphate (CaP) is a commonly used mineral in biological systems that easily permeates cell membranes and is easy to create when mixed with PAA. In this study, sufficient numbers of transformants were obtained for three out of the four gRNAs using the RNP/NP complex. Without using selectable markers and through random isolation, we achieved transformation rates of 6% for gHD1-1 and 3.5% for gHD1-2 and gHD2-2, providing sufficient efficiency for further research. These results indicate that the CaP nanoparticles effectively protect the sgRNA and permeate the cell membrane of the protoplasts, releasing an adequate amount of RNPs into the cytoplasm.

While the use of CaP nanoparticles showed promise in this study, it is crucial to acknowledge the broader implications of RNP-based gene editing in mushrooms. Studies on *Schizophyllum commune* [27] and *Flammulina velutipes* [50] have reported the successful application of RNP-mediated gene editing, highlighting its potential across various mushroom species. However, differences in editing efficiency among species underscore the need for the species–specific optimization of delivery platforms. Additionally, the compatibility of CaP nanoparticles with diverse fungal systems and the potential to combine this approach with other RNP delivery methods, such as gold nanoparticles or cell-penetrating peptides, should be explored to further enhance efficiency and reliability. Addressing these challenges will enable the broader application of RNP-mediated gene editing and contribute to advancements in fungal biology and strain development.

During the analysis of the transformants, an unexpected phenomenon of multinucleate monokaryons was observed during attempts to perform DNA-free RNP-based gene editing in *L. edodes*. Despite using the monokaryotic strain SJ705-13 for transformation, half of the obtained transformants were identified as multinucleate monokaryons. Hoechst 33258 staining revealed 2–3 nuclei in a single hyphal cell of the transformant HD1-1_78 (Figure S1). This finding contrasts with plasmid-based methods, where multinucleate monokaryons are rarely reported, highlighting a potential limitation of the DNA-free RNP editing approach. These results suggest that cellular dynamics related to nuclear distribution during protoplast transformation might contribute to this phenomenon. For instance, the multinucleate nature of fungal cells and interactions with the RNP delivery system could be factors. Addressing this issue may require the optimization of nanoparticle composition, RNP concentration, and delivery conditions.

Furthermore, the analysis of the *hd* genes and downstream-related genes such as *clp1*, *znf2*, and *priA* in the transformants suggests that the role of the HD1 subunit in the dimeric HD protein is relatively minor. When HD1-1_15 was crossed with the wild-type strain SJ710 (A1), the expression of the related genes in the dikaryon increased, and the mating process showed no significant differences as compared to the wild type. In contrast, the HD2 transformants exhibited inhibited dikaryon formation and a significant reduction in the expression of related genes as compared to HD1, underscoring HD2’s critical regulatory role in mating. Notably, the observed reduction in the expression of *clp1*, *znf2*, and *priA* in the *hd2* transformants highlights the central importance of HD2 in regulating mating processes at the molecular and cellular levels. These findings indicate that HD2 is essential for both the initiation and progression of mating events, with HD1 and HD2 working together to coordinate the fine-tuned regulation of these processes.

Interestingly, when gHD2-2 RNP was introduced into the HD1-1_15 transformant, no transformants were obtained, and the HD2 transformants failed to form dikaryons. This result suggests that, while the loss of HD1 alone does not critically disrupt mating, the loss of HD2 or the simultaneous loss of HD1 and HD2 could have fatal consequences for the survival of *L. edodes*. This observation supports the hypothesis that the HD dimeric protein plays a central role in controlling critical mating-related mechanisms, such as gene expression regulation and nuclear migration, and provides new insights into the distinct and interdependent roles of HD1 and HD2.

These findings go beyond a simple enumeration of results by providing a deeper understanding of the biological functions of HD1 and HD2 and a comprehensive evaluation of the strengths and limitations of DNA-free, RNP-based gene editing. Future efforts to optimize this technology should consider the unique cellular characteristics of multinucleate fungal systems during transformation to improve the generation of monokaryotic transformants.

## 5. Conclusions

In this study, high gene-editing efficiency was achieved using the CRISPR/Cas9 system based on RNP/NP complexes. By encapsulating RNP with calcium phosphate and polyacrylic acid, the transport efficiency into *L. edodes* protoplasts was maximized. This approach successfully targeted and edited the mating type genes *hd1* and *hd2* in *L. edodes*, followed by an analysis of the transformants. The results revealed that the loss of the *hd1* gene did not significantly affect growth, whereas the loss of the *hd2* gene completely inhibited mating. Additionally, it was indirectly confirmed that the simultaneous loss of both *hd1* and *hd2* genes could have fatal consequences for survival. Notably, when attempting DNA-free RNP transformation without selectable markers, multinucleate monokaryons accounted for half of the total transformants due to the multinucleate nature of *L. edodes* monokaryons. While this study highlights challenges that need to be addressed in RNP-based gene editing, it also demonstrates the potential of DNA-independent gene editing technology. The gene-editing efficiencies achieved using the RNP/NP complex were 6% for *gHD1-1* and 3.5% for both *gHD1-2* and *gHD2-2*. Compared to previous studies of species such as *Schizophyllum commune* [27] and *Flammulina velutipes* [50], which reported editing efficiencies of 5–7% and 4–8%, respectively, the results are comparable. However, unlike those studies that relied on selectable markers either through insertion or target-based selection, this study employed a completely selection-free approach. As a result, this method demonstrates greater potential and suggests higher actual editing efficiency in practical applications.

## Data Availability

The original contributions presented in this study are included in the article/Supplementary Materials. Further inquiries can be directed to the corresponding author(s).

## Supplementary Materials

The following supporting information can be downloaded at: https://www.mdpi.com/article/10.3390/jof10120866/s1: Figure S1: Observation of multiple nuclei present in a single hyphal cell; Table S1: primer set.
