# Supplementary material for: Effect of a Mating Type Gene Editing in Lentinula edodes Using RNP/Nanoparticle Complex
Source: J Fungi (Basel). 2024 Dec 13;10(12):866. doi: 10.3390/jof10120866 (PMC11677008; doi:10.3390/jof10120866)
Supplement: Supplementary file 1 [file jof-10-00866-s001.zip › jof-3325367-supplementary.pdf]

Table S1. Primer used in this study

Name	Direction	Nucleotide sequence (5' to 3′)
HD1 for cleavage	F	CATGATAGAGCCCACCGACG
	R	GAACGCCAACCCCATCCCC
HD2 for cleavage	F	CACCAGAAATTAAGGCAAGTTC
	R	TGTTGTCCGTGACCTTGAC
A1 marker	F	GATCTCGACGTTCGCCTAAG
	R	GTTGCTCTTCGAGAGGTAG
A5 marker	F	CAATCGTCTACTCACCTCC
	R	GCGAGGGTGCTTCTGATTC
CLP1	F	CCGAGTACTTCGCAACCAAC
	R	GTCGACCTGTGTTACCACGC
ZNF2	F	CAGCAACACCAATCCGACCG
	R	GAGTGCTCCGATTACCTTTG
priA	F	CCGTCATCGAAGTTGTTGTC
	R	GCACTTGCCGTTGCAAACTG
HD1 for RT	F	CACTCTATCGTCGAAACTGC
	R	CTACCAAGTCCCTCCTCCTC
HD2 for RT	F	CGGAGGTGCCGAAGTATAAG
	R	CTTATCCCGATAAAGCATCC
β-tubulin	F	GACCGTATGATGTGCACGTAC
	R	CACAAGATGGTTGAGGTCACC

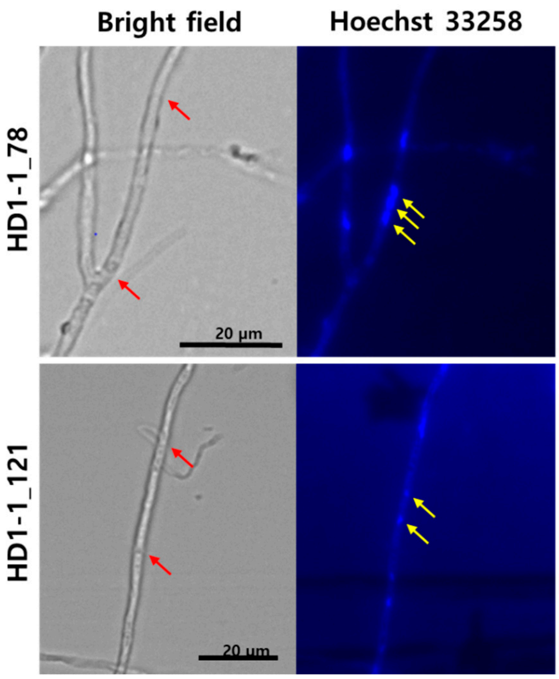

Figure S1. Observation of multiple nuclei present in a single hyphal cell. The Red arrow represents septa, The Yellow arrow represents Nucleus.
